# Draft Genome Sequence of Lactobacillus jensenii Strain UMB7766, Isolated from the Female Bladder

**DOI:** 10.1128/MRA.00392-20

**Published:** 2020-05-14

**Authors:** Taylor Miller-Ensminger, Alan J. Wolfe, Catherine Putonti

**Affiliations:** a Bioinformatics Program, Loyola University Chicago, Chicago, Illinois, USA; b Department of Microbiology and Immunology, Stritch School of Medicine, Loyola University Chicago, Maywood, Illinois, USA; c Department of Biology, Loyola University Chicago, Chicago, Illinois, USA; d Department of Computer Science, Loyola University Chicago, Chicago, Illinois, USA; Indiana University, Bloomington

## Abstract

Lactobacillus jensenii is a beneficial and prominent community member within both the vaginal and female urinary microbiota. As more genomes for L. jensenii strains are made publicly available, we gain more knowledge about this beneficial community member. Here, we present the draft genome sequence of L. jensenii UMB7766, which was isolated from a urine specimen from a catheterized female patient with recurrent urinary tract infections.

## ANNOUNCEMENT

Lactobacillus jensenii is a dominant member of both the vaginal and female urinary microbiota of healthy individuals (1, 2). L. jensenii acts as a protective species within both niches, showing bactericidal effects against pathogens. Notably, in the urinary tract, L. jensenii has been shown to reduce the growth of uropathogenic Escherichia coli (3), the most common cause of infrequent urinary tract infections (4–6). Last year, our group released 11 L. jensenii genomes (7), raising the number of characterized L. jensenii strains from the bladder to 13. To add to this collection, we present findings for L. jensenii UMB7766, which was isolated from a urine specimen from a catheterized female patient with recurrent urinary tract infections.

The sample was collected from a patient at the Women’s Pelvic Medicine Center at the University of California, San Diego, California, in August 2017 as part of an institutional review board (IRB)-approved study (University of California, San Diego, IRB approval number 170077AW). Urine was cultured using the expanded quantitative urine culture (EQUC) method (8). The genus and species for L. jensenii UMB7766 were determined by matrix-assisted laser desorption ionization–time of flight (MALDI-TOF) mass spectrometry following the protocol detailed previously (8). The strain was streaked onto a Columbia nalidixic acid (CNA) agar plate with 5% sheep blood plate (catalog number 221353; BD). The plate was incubated at 35°C in 5% CO_2_ for 48 h. A single colony was selected and grown in liquid MRS medium supplemented with 1 ml/liter Tween 80. DNA was extracted using a blood and tissue kit (catalog number 69504; Qiagen), following the manufacturer’s protocol for Gram-positive organisms, and quantified using a Qubit fluorometer. Library preparation and sequencing were performed by the Microbial Genome Sequencing Center at the University of Pittsburgh. Library preparation was performed using Illumina Nextera chemistry, with sequencing on the Illumina NextSeq 550 platform (2 × 150-bp reads). Sequencing produced 3,314,892 pairs of reads. Raw reads were trimmed using Sickle v1.33 (https://github.com/najoshi/sickle) and assembled using SPAdes v3.13.0 with the only-assembler option for k values of 55, 77, 99, and 127 (9). The coverage was calculated using BBMap v38.47 (https://sourceforge.net/projects/bbmap). The genome was annotated using the NCBI Prokaryotic Genome Annotation Pipeline (PGAP) v4.11 (10). The CRISPRCasFinder Web server was used to detect CRISPR arrays (11), and Mash was used to identify similar genomes (12) through the PATRIC v3.6.3 Web server (13). Unless specified otherwise, default parameters were used for all software tools.

The L. jensenii UMB7766 genome is 1,710,714 bp long. It was assembled into 41 contigs with an *N*_50_ value of 68,917 bp and 475× coverage. At 34.32%, the GC content of this strain is similar to that of other L. jensenii strains available in GenBank. PGAP identified 1,637 genes, with 1,529 protein-coding sequences. Furthermore, it identified 54 tRNA sequences, 3 5S rRNA sequences, 1 16S rRNA sequence, and 1 23S rRNA sequence. A type II CRISPR system was identified, and this strain has 1 CRISPR array with 15 spacers. The complete genome of L. jensenii UMB7766 was also compared to those of all publicly available strains of this species, including other isolates from the urinary tract and isolates from the vaginal and gut microbiota. This comparison identified L. jensenii UMB3442 (whole-genome sequencing GenBank accession number VYVY00000000), which was also isolated from the female urinary microbiota, as having the closest related genome. This finding suggests that L. jensenii strains of the urinary tract may more closely resemble each other than those of the vaginal microbiota.

### Data availability.

The whole-genome sequencing project for L. jensenii UMB7766 has been deposited in GenBank under the accession number JAAUWC000000000. The raw sequencing reads have been deposited in GenBank under the accession number SRR11441033.
